# Evaluation of pediatric dentists’ approaches to oral health literacy: Nevra Karamüftüoğlu

**DOI:** 10.1093/eurpub/ckac129.727

**Published:** 2022-10-25

**Authors:** N Karamüftüoğlu, D Atabek, A Uğraş Dikmen, F Semanur Korkmaz Öner, S Özkan

**Affiliations:** 1 Dentistry Faculty, Pedodontics, Cyprus International University, Lefkoşa, Cyprus; 2 Dentistry Faculty, Pedodontics, Gazi University, Ankara, Turkey; 3 Public Health, Gazi University, Ankara, Turkey

## Abstract

**Aim:**

In the study;it is aimed to determine the knowledge,attitudes,behaviors and educational needs of pediatric dentists about oral health literacy(OHL).

**Methods:**

The study was carried out with 100 pediatric dentists across Turkey between 20/06/2021-20/07/2021 on the online platform.In the questionnaire form;there are 14 descriptive,25 approaches to OHL in a 5-point Likert format and 8 questions about communication barriers in individuals with insufficient OHL.

**Results:**

The mean age of the participants was 34.4±7.9 years.92.0%(n = 92) of the participants did not receive OHL training.63%(n = 63) of the participants stated that it is necessary to carry out studies to improve OHL and to develop programs.The mean OHL approach scores of the participants were 3.81±0.61;the mean score of evaluating communication barriers in individuals with insufficient OHL level was 4.06±0.62.60%(n = 54) of the participants think that the health information in the media is very effective in misleading patients.As the age of the participants increased,the OHL approach score increased(r = 0.204;p=0.042).As the number of daily outpatient clinics of the individuals participating in the research increases,the evaluation scores of communication barriers decrease(r=-0.304;p=0.004).Among the participants,those who work in private institutions,those who know their rights while providing oral health services and those who are satisfied with the profession have higher scores for evaluating the OHL approach,the difference is statistically significant(p < 0.05).

**Conclusions:**

As a result,positive benefits will be provided to the professional satisfaction of pediatric dentists and the quality of oral health services with in-service trainings to be organized before and after graduation on the evaluation of communication barriers in individuals with inadequate OHL approach and level.

**Key messages:**

• Oral health literacy is a new concept and is becoming increasingly important in dentistry.

• The importance of oral health literacy in pediatric dentistry is that both children and parents are affected.

